# On the Toxical and Other Properties of “A Substance Analogous to Gun Cotton”*Extracted from an Inaugural Dissertation Recently Presented to the Faculty of Jefferson Medical College.

**Published:** 1849-05

**Authors:** William F. Jackson

**Affiliations:** Brunswick, Maine


*On the Toxical and other Properties of "A substance analogous to Gun Cotton."** By WILLIAM F. JACKSON, M. D., of Brunswick, Maine.

During the last few years there have been several very valuable discoveries, but perhaps no one of them has created more excitement among chemists of every country than that of gun cotton by Schönbein in 1846. No sooner had it been announced by him than various series of experiments were instituted, some of which resulted in the discovery of new substances, the value of which time alone can show. It is my purpose to call your attention to one of these substances discovered by Mr. Sobrero, a distinguished Spanish chemist, and announced by him in the "Comptes Rendus" for February 1847. The article was copied into the "London Chemical Gazette," and attracted the attention of Mr. Morris Davis, an operative chemist of this city,† who was the first in this country to prepare and experiment upon it. It has also been prepared and experimented upon by one or two others: and all concur in saying that it is a most powerful excitant, and capable of producing the most injurious results even when taken in very minute doses. How valuable it may prove as a therapeutical agent I am not prepared to say; but when we consider how many of our most valuable remedies are the most virulent poisons, we have a right to infer that this may not be without its uses. Should it not prove valuable as a medicinal agent, it is certainly, in a physiological point of view, worthy of an extended and careful examination.

*Mode of Preparation.*—The mode of preparing this substance given by Mr. Sobrero is as follows: "When a mixture of 2 vols. of sulphuric acid of 1.83 and 1 vol. of nitric acid of 1.43 is poured into syrupy glycerine, a very lively oxidation ensues, the product of which I have not ascertained; if, on the contrary, the above mixture of the two acids is placed in a freezing mixture, and glycerine poured into it, agitating to avoid all elevation of temperature, the glycerine quickly dissolves without any perceptible reaction: if the mixture be now poured into water, an oily substance heavier than water subsides to the bottom of the vessel;

* Extracted from an Inaugural Dissertation recently presented to the Faculty of Jefferson Medical College.

† Philadelphia.

when it is washed with a considerable quantity of water, to free it entirely from acids, without any loss, as it is quite insoluble in that menstruum. When well washed, it is wholly dissolved in alcohol, and precipitated again by water, or dissolved in ether, and the solution left to spontaneous evaporation, when it is obtained in a state of perfect purity. It is readily freed from water by keeping it for a few days in vacuo over sulphuric acid.

"In this state the body has the appearance of olive oil coloured slightly yellow; it has no odour; its taste is sweet, pungent and aromatic; but in making this experiment great precaution should be used, for a very minute quantity held upon the tongue produces a violent headache for several hours. The effect upon the human body was experienced by several persons in my laboratory, and I have frequently felt its effects myself."

As far as my experience goes, it is not necessary that the acids shall be of the specific gravities given above, for in my experiments I used the Nordhausen sulphuric acid of 1.86 and nitric acid of 1.48, and succeeded perfectly. I am well satisfied that it matters little what the strength of the acid may be, provided that it is *strong enough*. The greatest difficulty which I met with was from the glycerine. In my first experiments I failed in every instance, in consequence (as I think) of the oxide of lead which had not been precipitated, and of the large amount of water which was combined with the glycerine. After having got rid of the lead by precipitation, and having evaporated the glycerine to one half, I found no difficulty, though great care was necessary to prevent the rising of temperature. Whenever the temperature rises above a certain point, an intense chemical action takes place, and a dense cloud of nitrous acid fumes is thrown off. Whenever this *improper* action takes place, the capsule will be found to contain a substance resembling molasses both in colour and consistence, soluble in alcohol and water, and having a sour and bitter taste, and an odour resembling somewhat that of burnt sugar. In one or two instances, the diminution of the nitrous acid fumes was preceded by a distinct explosion, and in one instance the contents of the capsule were thrown in all directions.

The acids are to be poured into the capsule, to be agitated for a few moments so as to mix them intimately, and allowed to stand until they are cooled down to the proper point. The prepared glycerine is then to be added *slowly*, and the mixture must be *constantly* stirred. It gradually becomes thicker, and soon resembles honey both in colour and consistence. It is now to be poured into water, and the substance sought for will, after a short time, be found in small opaque globules, resembling very much the globules of oil in an emulsion, at the bottom of the vessel. After having been thoroughly washed it may be freed from water by means of the sulphuric acid, as recommended by Mr. Sobrero, or by means of a test tube having a very small aperture at the bottom; the substance being heavier than water, will of course pass off first. Care must be taken to prepare it only in small quantities, as explosions are very liable to occur, causing, of course, loss both of time and materials.

What the precise chemical nature of this substance may be, I am at present unable to state. My first impression was, that it was a nitro-sulphate of glycerine, but I am now convinced that the glycerine undergoes a decomposition, and that a new substance is formed from the combination of its elements with those of the acids.

*Toxical effects.*—On account of the unpleasant effects which this substance produces, it is almost impossible to find persons willing to make experiments with it, and even *medical students* are not over anxious to "gratify their curiosity." I have endeavoured, while making my experiments, to avoid, as far as possible, all extraneous influences, and to make allowances where such might occur; but the position of a medical student is certainly not the one best calculated to conduct a series of experiments requiring so much care and precaution.

In the majority of trials I have used one-third or one-half of a drop of the alcoholic tincture, but occasionally I have increased the dose when I wished to get more decided effects. It is safer, however, to use small doses, and repeat if necessary, for the effects produced by large doses are both unpleasant and dangerous.

*February 18th.*—Pulse at sixty-five beats per minute, and body free from any unpleasant sensations—took one-third of a drop of the alcoholic tincture. In thirty seconds the pulse had risen to eighty beats per minute. Disagreeable sensation of fulness in the forehead. In thirty seconds more the pulse was at ninety, and the pain in the forehead quite severe. No other symptoms were developed, and in half an hour the pulse had returned to seventy beats per minute, and the pain was entirely gone. I then repeated the dose. The pulse rose as before to ninety-five, the pain in the head became quite severe, and a sensation as if the eye-balls were being pushed out was produced. When the symptoms began to abate I again repeated the dose. The pulse *instantly* rose to one hundred and twelve. Pain in the head intense. Eyes protruding and injected, scintillations as in head affections caused by disordered stomach. Fulness at the base of the brain, and violent throbbing of all of the arteries of the head and neck. Laboured action of the heart with a peculiar sense of oppression. I went into the open air, and in a short time the most striking symptoms disappeared, leaving only a sense of languor and an unpleasant sensation about the heart.

*February 20th.*—The above experiment was repeated but no new symptoms were produced. The disagreeable feelings about the heart, however, were somewhat aggravated, but soon passed off.

*February 22d.*—Having recovered entirely from the former experiments, I determined to make another trial, using a larger dose, however, than I had done heretofore. The pulse was at sixty-six beats per minute and perfectly regular. I took *one drop* of the tincture, and the pulse in less than a minute rose to one hundred and twenty-four beats per minute. It was hard, distinct, and almost incompressible. The heart laboured violently and a lancinating pain passed from the region of the heart to the back between the shoulders. The pain in the head was almost unendurable, particularly in the forehead, and the disagreeable feelings at the base of the brain were far more severe than I had yet known them. Eyes were injected and seemed to protrude, the pupils were somewhat dilated. Flashes of light were almost continuous and vision of course indistinct. The tongue and mouth burned, and the former felt swollen and raw, and was affected by spasmodic twitchings. Respiration was not impeded, though there was a sensation of constriction about the chest. In two minutes the pulse had fallen to one hundred beats per minute, and was decidedly intermittent. The symptoms gradually disappeared, and in half an hour no positively disagreeable sensations remained.

The same sensation of weariness and oppression about the heart was noticed as in former trials.

I have also the results of several other experiments, but as they do not differ materially from those given above, I do not think it necessary to detail them.

*February 24th.*—Experiment upon a cat. In attempting to put this preparation, in substance, into her mouth I accidentally touched the glass rod upon the end of her nose ; immediately her head was thrown backwards upon her neck and maintained there. Saliva flowed freely from her mouth, which was open and the tongue protruding. The eyes were glassy and fixed, and the pupils very much dilated. She walked *backwards*, but with great difficulty, as her limbs were extremely rigid. The pulse was very rapid, but as I had neglected to count it before commencing the experiment, I could not tell how much it had been affected. As soon as she began to recover, I put about *three* drops of this substance into her mouth. There was no effort to move after a few seconds ; the limbs were perfectly paralyzed and rigid. The contractions of the heart could not be counted, though they could be distinctly felt. Respiration was very difficult and rapid. The eyes actually stood out of the head, and the iris was scarcely visible. In about two minutes from the time in which I put the poison upon her tongue she ceased to breathe, though the heart beat a few seconds longer. Spasmodic contractions of the legs occurred for some time after both respiration and circulation had ceased.

I intended to make an extended series of experiments upon animals, but circumstances beyond my control have hitherto prevented my doing so. At some future time, however, I hope to be able to give a more satisfactory account of the effects of this substance ; sufficient has been done, however, after having made all due allowance, to show the great power, and the rapidity of its action.

In its toxical effects it resembles very nearly the aconitum napellus, but is far more rapid in its action, if not more fatal in its results.

Whether or not this substance will prove valuable in the treatment of disease remains yet to be seen, but I am well convinced that it is destined to occupy no mean position in the list of therapeutical agents.

